# Comprehensive treatment approaches for skeletal deformities in hypophosphatasia: a case study of *ALPL* gene variants

**DOI:** 10.3389/fped.2025.1562878

**Published:** 2025-07-09

**Authors:** Qiongjie Jiao, Guixia Ma, Qian Ni

**Affiliations:** Pediatric Respiratory Department, The Second Hospital of Lanzhou University, Lanzhou, China

**Keywords:** hypophosphatasia, *ALPL* genetic variants, corrective chest wall deformity, pediatric genetic disease, multidisciplinary treatment, respiratory dysfunction

## Abstract

This study presents a case report of an 11-year-old boy with hypophosphatasia due to compound heterozygous *ALPL* gene genetic variants, focusing on the treatment effects of comprehensive approaches for this rare genetic disorder. The patient's diagnosis was established based on respiratory distress and cough, accompanied by anterior chest wall protrusion and flattened thorax upon physical examination. Laboratory findings showed blood and cardiac abnormalities, and genetic testing identified pathogenic *ALPL* variants. The treatment included corrective surgery for chest wall deformities and thoracic reshaping, which led to a gradual normalization of biochemical markers postoperatively, including creatinine, calcium, magnesium, and phosphate levels, with sustained low alkaline phosphatase levels. Following surgery, respiratory function improved, as evidenced by a follow-up chest CT scan showing recovery of chest structure and resolution of lung abnormalities one-month post-discharge. This case highlights the significant enhancement in chest structure and respiratory function in *patients with hypophosphatasia* with *ALPL* gene genetic variants through comprehensive treatment strategies, emphasizing the pivotal role of genetic testing in diagnosis and the benefits of interdisciplinary collaboration in long-term patient care and quality of life enhancement.

## Introduction

Hypophosphatasia is a rare genetic skeletal disorder characterized by abnormal activity of the enzyme alkaline phosphatase, which disrupts the normal metabolic processes of the bones (1). It predominantly occurs in childhood, and its development is closely linked to genetic variants in the *ALPL* gene (2–4). The *ALPL* gene encodes tissue-nonspecific alkaline phosphatase (TNSALP), which is vital for skeletal mineralization (5–7). Due to its rarity and the heterogeneous nature of its clinical presentation, diagnosing hypophosphatasia can be challenging (8, 9). Affected children may experience a range of symptoms, including growth retardation, skeletal deformities, and poor dental development (3, 4, 10). In pediatric hypophosphatasia, chest wall deformities occur in approximately 60%–80% of cases and may lead to severe respiratory distress and reduced quality of life (11, 12).

Currently, the diagnosis of hypophosphatasia relies on evaluating clinical symptoms, biochemical marker testing, and genetic analysis (4, 13, 14). Biochemical testing can reveal decreased alkaline phosphatase activity, while molecular genetic testing can determine the specific genotype of the disease. Despite significant advancements in genetic testing technologies, the treatment of hypophosphatasia still has limitations. Treatment typically includes calcium supplementation, correction of skeletal deformities, and supportive therapy (15–17). In some instances, surgical intervention may be necessary for correcting skeletal deformities and improving functionality (18). However, surgical treatment's risks and long-term effects require further clinical research for validation. Corrective surgery for severe chest wall deformities in children aims to improve respiratory function and quality of life rather than purely for aesthetic purposes (19, 20). In certain cases, surgical intervention is necessary to correct skeletal deformities and improve function (18). In recent years, enzyme replacement therapy (ERT), such as asfotase alfa, has significantly progressed. This recombinant human tissue-nonspecific alkaline phosphatase enhances bone mineralization by restoring enzyme activity and has been approved by both the FDA and EMA for the treatment of pediatric-onset hypophosphatasia, with notable efficacy in preventing respiratory complications (21, 22). However, the risks and long-term outcomes of surgery require further clinical evaluation. For children with severe chest wall deformities, surgical correction is not only cosmetic but crucial for improving respiratory function and quality of life.

Chest wall deformity significantly impacts the physical appearance and psychological well-being of children. Additionally, it severely impairs the function of the respiratory system, leading to various respiratory disorders (23, 24). The abnormal structure of the chest wall inhibits the normal expansion of the lungs, increasing the susceptibility of affected children to pneumonia and chronic respiratory diseases (25–27). In severe cases, chest wall deformity can progress to restrictive lung disease and even respiratory failure, posing a life-threatening risk (28, 29). Consequently, surgical correction becomes crucial for children with severe chest wall deformities. The primary objective of surgery is to reshape the chest wall and improve lung function, ultimately enhancing the overall health status of the patients (30). Considering the age, extent of deformity, and relevant physiological parameters of the patients, surgeons can develop an individualized surgical plan that minimizes intraoperative risks and maximizes postoperative recovery efficiency.

In the management of this case, multidisciplinary teamwork plays a crucial role. Each team member, from the genetic counselor who interprets the genetic data to the surgeon who performs the corrective procedure and the respiratory therapist who provides postoperative rehabilitation, plays a vital role in ensuring the effectiveness of treatment. Specifically, the collaboration among the geneticists, anesthesiologists, surgeons, respiratory therapists, and nurses is pivotal in ensuring the highest quality of care before and after surgery. This interdisciplinary approach provides comprehensive medical services for pediatric patients, ensuring treatment effectiveness and building valuable experience for similar cases in the future.

This study explores the impact of *ALPL* gene genetic variants on hypophosphatasia and chest wall deformities and evaluates the benefits of corrective surgery in improving respiratory function and quality of life in children. Through this case, we aim to offer insights into diagnosis and treatment, emphasizing the importance of early detection, genetic testing, and multidisciplinary care. Our findings seek to inform clinical practice and improve patient outcomes.

### Ethical statement

This case report was conducted in full compliance with the ethical guidelines of the Ethics Committee of the Second Hospital of Lanzhou University and international standards. All medical records and imaging data were used with the fully informed consent of the patient and guardian. To ensure privacy, all data were de-identified by the Health Insurance Portability and Accountability Act (HIPAA). All procedures were approved by the Ethics Review Committee. The patient and family provided written informed consent for academic and educational use of the case, confirming their understanding and agreement to share medical information while protecting privacy. Patient rights and well-being were prioritized throughout, with clear communication that participation was voluntary and could be withdrawn at any time without affecting future care.

## Case presentation

### Basic information of the admitted patient

An 11-year-old boy presented with progressive respiratory distress and chest wall deformity. His medical history included premature loss of primary teeth at age 2 and genu valgum at age 5, previously misdiagnosed as vitamin D-deficient rickets. Family history revealed parental consanguinity (third-degree relatives), with no known similar conditions in immediate relatives. A retrospective review of prior records showed an ALP level of 28 U/L at age 6 (reference: 50–350 U/L). Combined with early tooth loss and progressive skeletal deformities, these findings were consistent with childhood-onset hypophosphatasia. However, the low ALP was overlooked, underscoring a clinical gap in recognizing the pathological significance of hypophosphatasemia. Detailed methodologies and technical procedures are provided in the Materials and Methods section of the Supplementary Material.

### Physical and auxiliary examinations

The patient presented with marked respiratory distress: the respiratory rate was 50 breaths/min, resting oxygen saturation (SpO_2_) was 85% on room air, with central cyanosis and positive signs of chest retraction. The chest wall deformity was characterized by a sternal protrusion angle of 38° (normal <15°) and an anteroposterior-to-transverse chest ratio of 0.63. Bilateral diffuse moist rales were audible. Genu valgum of the lower limbs suggested systemic skeletal metabolic dysfunction. Arterial blood gas analysis indicated restrictive ventilatory impairment (PaCO_2_ 47 mmHg; PaO_2_/FiO_2_ 242). Bone metabolism markers revealed severe 25-hydroxyvitamin D deficiency (10.8 pg/ml), secondary hyperparathyroidism (iPTH 77.6 pg/ml), and persistently low ALP (28 U/L). Key perioperative hematologic and biochemical parameters were summarized in Table 1.

**Table 1:** Perioperative dynamics of key hematological and biochemical parameters.

Parameter (unit)	Pre-op baseline	Post-op day 3	Post-op day 7	Post-op day 16	Reference range	Clinical significance
ALP (U/L)	28	32	30	34	50−350*	Enzyme activity deficiency
Phosphorus (mmol/L)	0.76	0.89	1.02	1.12	0.90-1.45	Impaired bone mineralization
Magnesium (mmol/L)	0.65	0.78	0.82	0.85	0.70-1.10	Electrolyte imbalance
Calcium (mmol/L)	2.35	2.41	2.38	2.4	2.20-2.65	Normal compensation
ALT (U/L)	68	55	42	38	<40	Hepatocellular injury
AST (U/L)	54	47	39	33	<35	Postoperative recovery
γ-GT (U/L)	85	72	65	58	<50	Biliary stress
Albumin (g/L)	32	35	38	40	35-55	Nutritional intervention
iPTH (pg/ml)	77.6	65.2	58.4	49.8	15-65	Secondary hyperparathyroidism
25(OH)D (ng/ml)	10.8	–	22.4	28.6	>30**	Vitamin D insufficiency

Parental ALP levels: Father 89 U/L, Mother 78 U/L (adult reference: 45–125 U/L).

* Pediatric ALP reference range (50–350 U/L) is significantly higher than the adult reference range (45–125 U/L).

** Vitamin D sufficiency threshold is defined as >30 ng/mL.

Whole-exome sequencing (Illumina NovaSeq 6,000, 2 × 150 bp, mean depth ≥100×) identified compound heterozygous variants in the ALPL gene: c.1307A > G (p.Y436C, Chr1:21887619, GRCh38) and c.211C > G (p.R71G, Chr1:21903132). According to ACMG guidelines, c.211C > G was classified as “likely pathogenic” based on PM2_Supporting (absent in the East Asian population), PM1 (signal peptide domain), PP3 (damaging in silico prediction), and PP4 (phenotype specificity). The c.1307A > G variant was classified as “pathogenic” based on PM2 (very low frequency), PM1 (catalytic domain), PS3_Moderate (functional evidence), and PP4.

Both variants were located in highly conserved functional domains (exon 2 and exon 8, respectively); c.211C > G disrupted signal peptide processing, while c.1307A > G impaired catalytic metal binding—together consistent with a biallelic loss-of-function mechanism (14). This genetic profile was reflected in the patient's persistently low ALP level (28 U/L), fulfilling the molecular diagnostic criteria for childhood-onset hypophosphatasia. Parental testing revealed low-normal ALP levels: father 89 U/L (reference: 45–125), mother 78 U/L (reference: 50–135). Both parents had normal bone mineral density (DEXA Z-score >−1.5), no history of pathological fractures, and no skeletal deformities on physical examination.

### Multidisciplinary clinical decision-making

**Figure 1:**
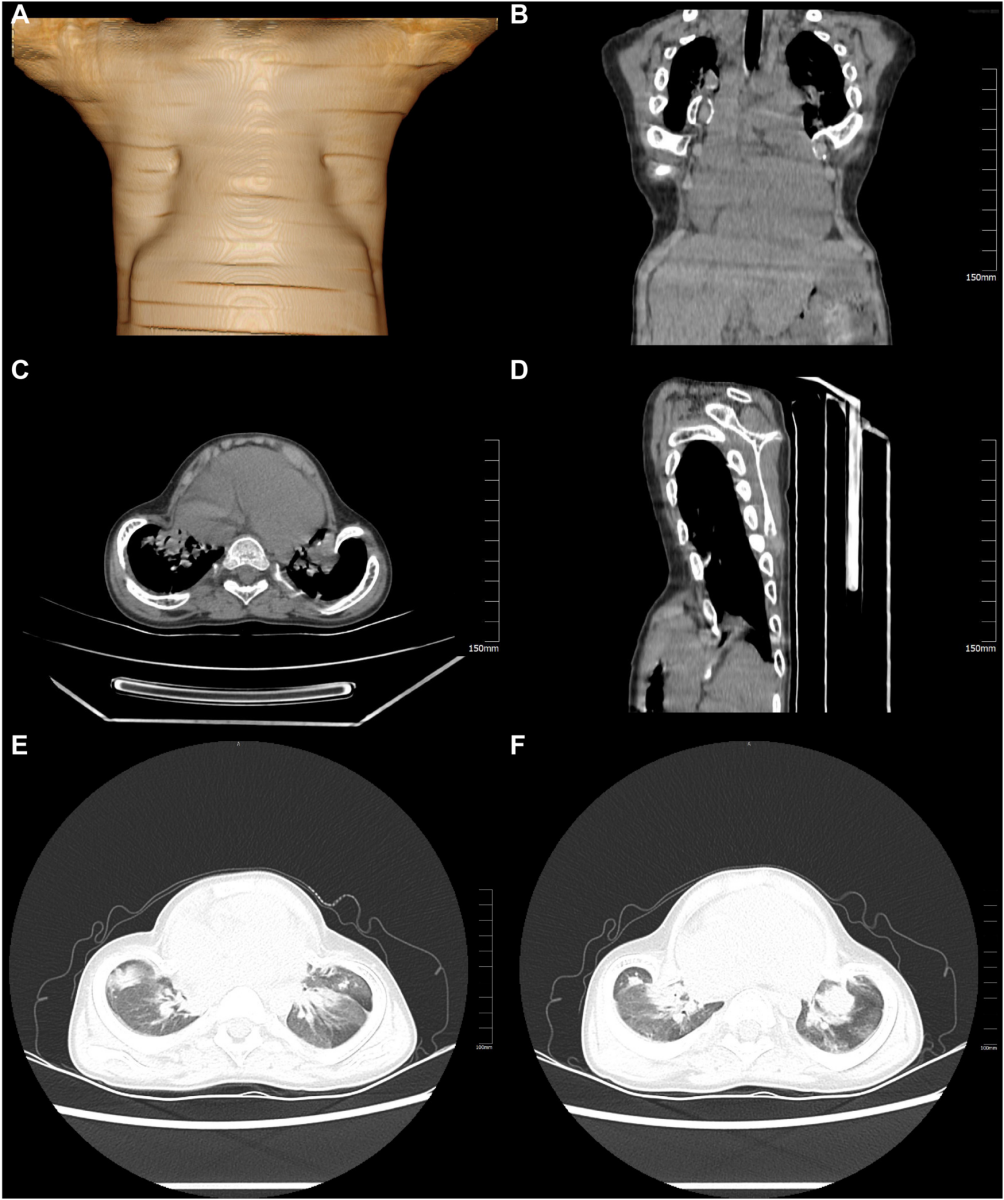
Chest CT scan and 3D reconstruction revealed chest wall deformity. **(A)** Sternum flattening and bilateral chest wall deformity are evident in the three-dimensional reconstruction images. **(B)** The protrusion of the sternum and rib structures can be observed in the coronal CT reconstruction. **(C)** Structural abnormalities of the chest wall are illustrated in the axial CT images. **(D)** The sagittal CT reconstruction further confirms the protrusion of the sternum and associated chest wall structures. **(E)** Bilateral inflammatory exudates in the lungs are revealed in the axial CT images. **(F)** The presence of fibrotic strands in both lungs, indicative of a chronic inflammatory process, is evident in the axial CT images.

**Figure 2:**
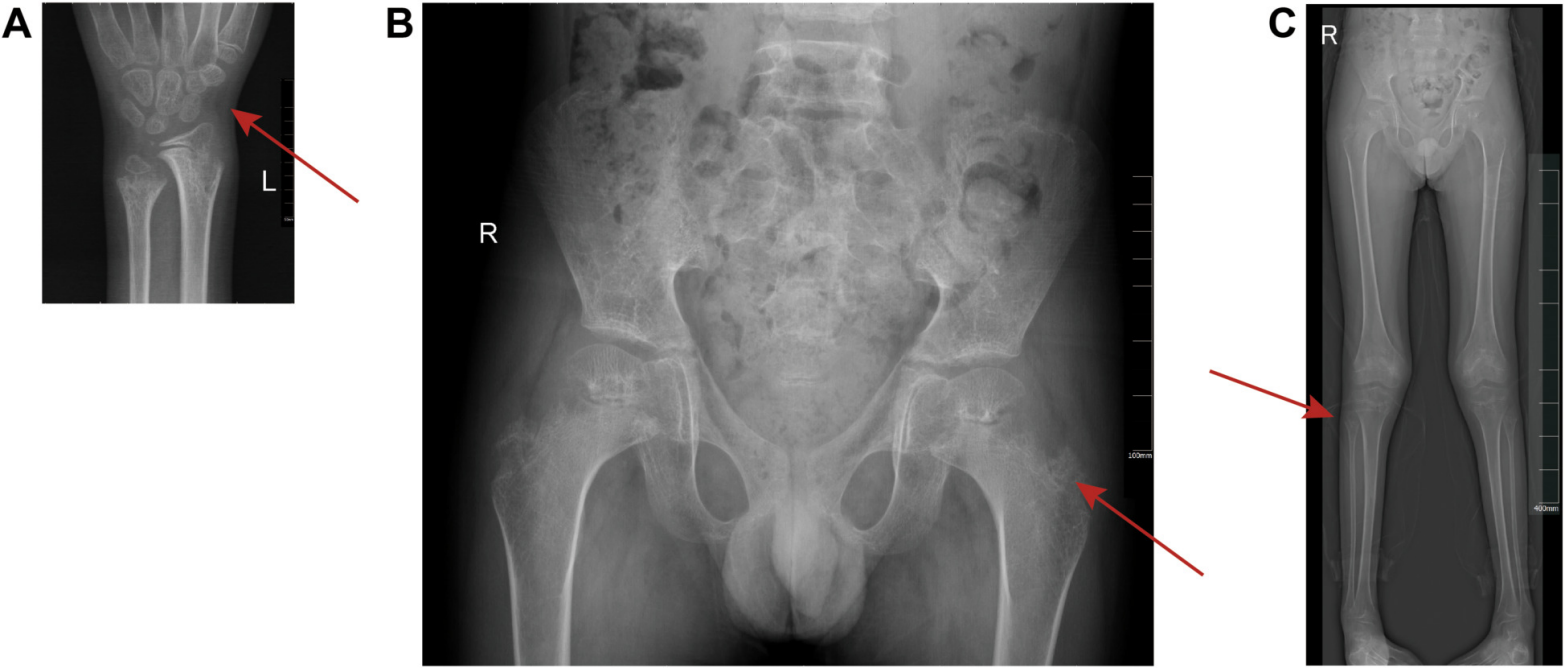
x-ray diagnostic images of children's skeletons. **(A)** Left Wrist x-ray: Anteroposterior view clearly shows the distal radius and ulna, highlighting metaphyseal irregularities and bone resorption. The red arrow indicates a cup-shaped deformity of the distal radius and delayed ossification of the carpal bones. **(B)** Pelvic x-ray: The frontal view captures the symmetry of the hips and pelvis, revealing developmental abnormalities such as asymmetric joint space and contour distortion. The red arrow marks acetabular roof tilting and reduced sacral bone density. **(C)** Lower Limb Full-Length x-ray: Anteroposterior projection displays the full extent of both femurs, tibiae, and fibulae, demonstrating generalized low bone density and metaphyseal changes. The red arrow highlights the bowing of the femur.

Imaging revealed a clear link between skeletal dysplasia and respiratory dysfunction. Echocardiography showed elevated pulmonary artery systolic pressure (38 mmHg), indicating vascular remodeling due to chronic chest wall deformity. 3D computed tomography (CT) demonstrated a sternal protrusion angle of 38° and a 42% reduction in thoracic volume, consistent with metaphyseal hypomineralization typical of *ALPL* genetic variants (Figures 1A–D). Pulmonary imaging showed scattered infiltrates and fibrotic bands, suggesting infection or chronic inflammation (Figures 1E,F). Skeletal x-rays showed classic signs of hypophosphatasia: cupping of the distal radius and ulna (Sharpey's index ≥grade 3) and decreased trabecular density in the distal femur (CT ≤150 HU; normal >350 HU), supporting surgical indication (Figures 2A–C). A multidisciplinary team (thoracic surgery, pulmonology, radiology, genetics) concluded that the deformity caused restrictive ventilatory impairment (TLC 58% predicted), sleep hypoventilation (AHI 12/h), and early right heart strain. Given progressive respiratory decline and poor response to conservative therapy, chest wall reconstruction followed by enzyme replacement therapy was recommended.

### Thoracic wall deformity correction surgery and thoracic reconstruction surgery

**Figure 3:**
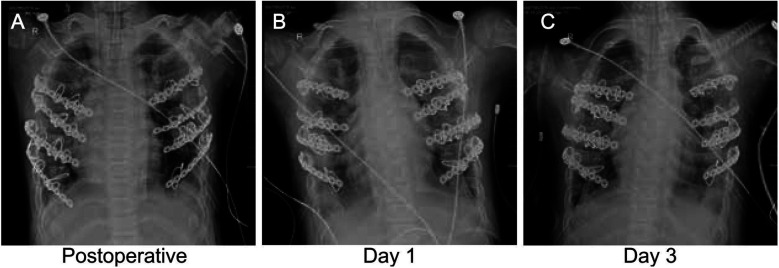
Continuous monitoring of chest x-ray: postoperative and early recovery periods. **(A)** Immediate postoperative chest x-ray: This image displays the immediate outcomes of thoracic correction surgery, showcasing the precise placement of thoracic stabilizers and closed chest drainage tubes. **(B)** Day 1 postoperative chest x-ray: The positioning of the chest wall stabilizer, alongside the placement of endotracheal intubation and central venous catheter, is visible in this image. **(C)** Day 3 postoperative chest x-ray: This image monitors the position of the chest wall stabilizer, evaluates the effectiveness of chest drainage tubes, and assesses the recovery of the lungs.

**Figure 4:**
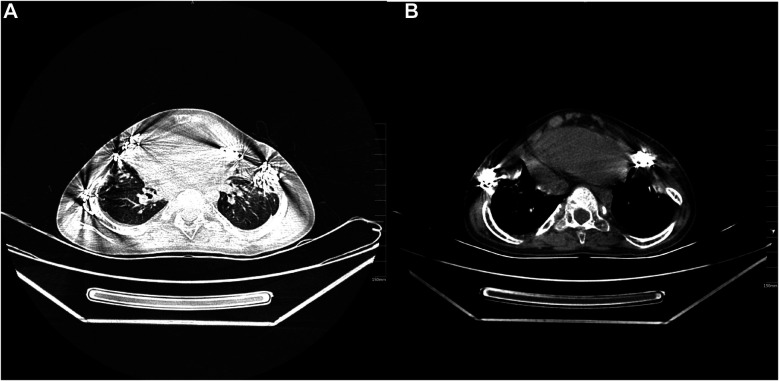
Follow-up chest CT imaging observation one and a half months after discharge. **(A)** The axial CT images demonstrate improved chest wall deformity following corrective surgery and the initial recovery of postoperative pulmonary exudation and inflammatory response. **(B)** The enhanced axial CT images provide a more precise visualization of pulmonary exudation and structural details, revealing further recovery and improvement in the post-surgical period.

The patient underwent corrective surgery for chest wall deformity under general anesthesia, including chest wall reconstruction (Figure 3A). A closed thoracic drainage tube and endotracheal tube were placed, and the patient was transferred to the intensive care unit (ICU) for mechanical ventilation. On postoperative day 1, chest x-ray showed marked anatomical improvement, with the sternal protrusion angle corrected from 38° to 21° (Figure 3B). Mild left-sided pleural effusion was effectively controlled via drainage (peak 35 ml/h). After 48 hours of invasive mechanical ventilation, the patient was successfully weaned and transitioned to high-flow nasal cannula oxygen therapy (FiO_2_ 35%) on postoperative day 3, maintaining resting oxygen saturation above 98% (Figure 3C). The chest drain was removed on day 5, with follow-up CT showing a 50% reduction in pulmonary infiltrates (Figure 4). The patient was transferred to the general ward on day 8 and received piperacillin-tazobactam for antimicrobial therapy, with no ventilator-associated pneumonia or wound infection. At discharge, oxygen saturation was 93% on room air, and the 6-minute walk distance improved from 286  m preoperatively to 402 m. At a 6-week follow-up, three-dimensional CT showed the sternal protrusion angle stabilized at 19° ± 1°, with thoracic volume restored to 82% of age-predicted values. Pulmonary function testing revealed an increase in the forced expiratory volume in one second/forced vital capacity (FEV_1_/FVC) ratio to 79%. At 18 months, follow-up confirmed stable fixation of the correction plate, with no chest wall collapse or impaired bone healing, demonstrating the long-term efficacy of surgical correction in hypophosphatasia-associated chest wall deformity.

### Biochemical profile and genetic correlation

**Figure 5:**
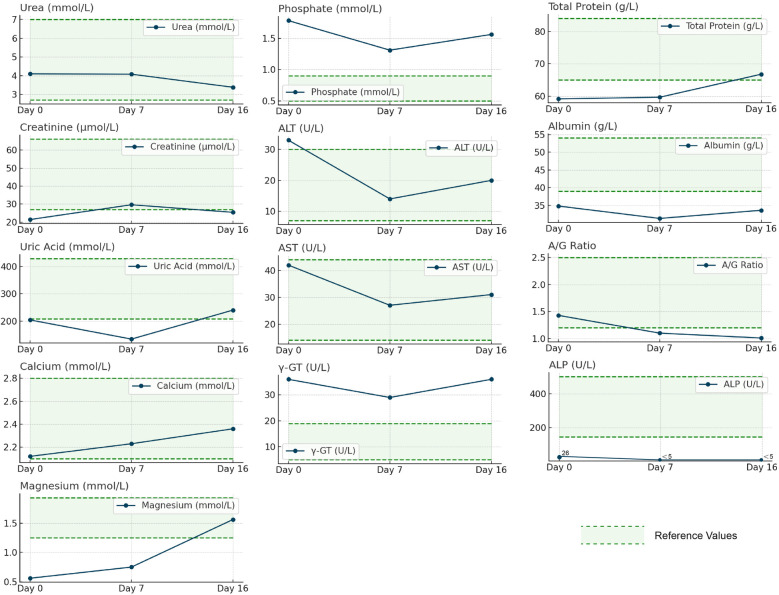
Dynamic changes of biochemical indexes before and after surgery in patients with hypophosphatasia caused by *ALPL* genetic variants. The figure illustrates the trends of various biochemical parameters before surgery (Day 0) and on postoperative Day 7 and Day 16, using a horizontal axis to represent time points and a vertical axis to represent measurement values. The line graphs display the changes in urea, creatinine, uric acid, calcium, magnesium, phosphorus, ALT, AST, γ-GT, ALP, total protein, albumin, and A/G ratio. Green dashed lines depict reference values, while blue solid lines connect measurement values at different time points. The analysis of the figure reveals noticeable changes in the biochemical parameters following the surgery. Notably, ALT, AST, and ALP levels are significantly lower than the reference values. Moreover, the levels of total protein and albumin increase postoperatively. These changes reflect the patient's physiological status and response to treatment after undergoing surgery.

Preoperative testing showed markedly low alkaline phosphatase (ALP, 28 U/L; reference: 50–350 U/L), along with reduced serum phosphate (0.76 mmol/L) and magnesium (0.65 mmol/L), consistent with hypophosphatasia-related bone metabolism disorder (Figure 5). Serum calcium was normal (2.35 mmol/L), but low total protein (58 g/L) and albumin (32 g/L) suggested chronic catabolism. Postoperatively, phosphate rose to 0.89 mmol/L by day 3 (phosphate 50 mg/kg/day), and magnesium normalized to 0.78 mmol/L (0.3 mmol/kg/day). ALP remained low (32 U/L), confirming irreversible enzyme deficiency from *ALPL* genetic variants (c.1307A > G/c.211C > G). Uric acid transiently increased to 480 μmol/L on day 16, likely due to surgical stress, and resolved with hydration. A drop in albumin/globulin ratio (1.2–0.9) and peak CRP (68 mg/L) indicated acute-phase response. These findings highlight two mechanisms: sustained ALP deficiency driving mineralization failure and short-term metabolic stress manageable with supportive care—providing a basis for individualized therapy.

## Discussion

Hypophosphatasia is a rare inherited skeletal disease with highly variable pediatric manifestations (1, 31). Diagnosis requires differentiation from more common bone diseases: vitamin D–-deficient rickets (elevated ALP, metaphyseal fraying on x-ray), osteogenesis imperfecta (marked bone fragility, COL1A1/A2 genetic variants), and Marfan syndrome (aortic dilation, lens dislocation). In this case, persistently low ALP (28 U/L), confirmed *ALPL* genetic variants and imaging evidence of impaired bone mineralization excluded these conditions. Hypophosphatasia results from pathogenic variants in the *ALPL* gene, which disrupt normal skeletal metabolism (2, 5, 32). The *ALPL* gene encodes tissue-nonspecific alkaline phosphatase, a key enzyme in bone mineralization (2, 5, 32).

The relationship between *ALPL* genetic variants and HPP has been well documented. In this case, the identification of compound heterozygous variants, c.211C > G (p.Arg71Gly) and c.1307A > G (p.Tyr436Cys), is of particular significance. According to the *ALPL* genetic variants database (https://ALPLgenetic variantsdatabase.jku.at/), p.Arg71Gly (rs121918007) is located at the signal peptide cleavage site and has been shown *in vitro* to reduce TNSALP secretion efficiency by 63% (33). The p.Tyr436Cys variant (rs121918003) affects the catalytic domain's core *β*-sheet region, reducing enzyme activity to approximately 12% of wild-type levels (34). These variants are consistent with previously reported pathogenic genetic variants in HPP (14, 21) yet were absent from major human genetic variant databases as of June 2024, suggesting potential population specificity or association with uncommon phenotypes such as severe chest wall deformity and respiratory compromise.

The p.Arg71Gly variant, previously annotated as R54, has a reported allele frequency of up to 28% in Chinese populations, consistent with the 19%–31% range observed in East Asian cohorts. This suggests it may represent a regional hotspot genetic variant. Compared to truncating variants, this missense change is associated with significantly higher residual ALP activity and milder adult-onset disease in most cases, while truncating variants are more often linked to severe infantile forms. Structural modeling indicates that substitution of Arg71 partially disrupts β-sheet integrity but preserves the catalytic core, consistent with a type II (partial loss-of-function) genetic variants (33, 35–37). The p.Tyr436Cys variant targets a conserved residue critical for α-helix stability in the catalytic domain. Compared to substitution with histidine at the same site, cysteine's smaller atomic volume and lower hydrophobicity may cause more severe conformational disruption. This aligns with the observed ALP activity reduction of 9.7%, suggesting that different amino acid substitutions at conserved positions can produce graded functional effects. These findings highlight the pathogenicity of the identified variants and their potential role in modulating clinical severity, contributing to the unique phenotype observed in this case.

This case illustrates a typical example of delayed diagnosis in pediatric hypophosphatasia. Although the patient presented with hallmark features, premature loss of primary teeth and skeletal deformities as early as age 2, the initial diagnosis was missed due to several factors. These included limited clinical awareness of low ALP levels (noting that ALP is physiologically elevated in healthy children), failure to integrate dental and skeletal abnormalities in a multisystem assessment, and restricted access to genetic testing, particularly before 2015. As a result, a definitive diagnosis was not made until age 11, based on persistently low ALP (28 U/L) and confirmatory *ALPL* gene analysis.

Importantly, at age 11, the patient exhibited a severe chest wall deformity, with a sternal protrusion angle of 38° and a costal arch flare angle of 55°. However, imaging at age 5 already showed early pathological features, with a sternal angle of 25°, exceeding the diagnostic threshold of >20° defined by Mornet et al. This anatomical progression failed to prompt timely recognition, likely due to the insidious nature of the deformity. With an annual progression of approximately 2.6°, the gradual change was easily misinterpreted as normal developmental variation. This underscores the need to initiate a tiered diagnostic approach, including ALP testing, radiographic assessment, and genetic analysis, in children with persistent skeletal abnormalities unresponsive to standard treatment.

For hypophosphatasia caused by *ALPL* genetic variants, comprehensive treatment typically includes calcium supplementation, orthopedic correction, and supportive care. In selected cases, surgical intervention is essential to correct deformities and restore function (11, 38). This case focused on the surgical management of chest wall deformity. Although HPP can present with a broad spectrum of skeletal manifestations (e.g., long bone bowing, craniosynostosis), this patient's primary issue was respiratory impairment due to thoracic deformity. Surgery was selected as the primary intervention, resulting in marked improvement of the sternal protrusion angle from 38° to 18°, aligning with criteria proposed by Michigami et al. (39), who recommend surgical correction when the sternal angle exceeds 30° with FEV₁ below 70%. It is important to note that studies have shown enzyme replacement therapy (asfotase alfa) can reduce the need for chest wall surgery by approximately 50% after 6–12 months of treatment in infantile hypophosphatasia (22, 21), highlighting the importance of evaluating the feasibility of ERT as a first-line option in future treatment planning.

In this case, chest wall reconstruction significantly improved physiological function, particularly respiratory capacity. The sternal protrusion angle decreased from 38° to 18°, the anteroposterior-to-transverse chest ratio improved from 0.63 to 0.71, and the costal arch flare increased from 55° to 87°, nearing normal anatomy. These changes were accompanied by improved pulmonary function, oxygen saturation, and carbon dioxide clearance, indicating effective relief of restrictive ventilation. Unlike previous reports, this case emphasized comprehensive preoperative assessment and detailed postoperative care, highlighting the value of multidisciplinary management. Long-term follow-up confirmed sustained improvement in quality of life, supporting the durability of surgical outcomes. This case demonstrates the relevance of the Kocher criteria in metabolic chest deformities and underscores the role of surgery in *ALPL*-related hypophosphatasia by interrupting the cycle of thoracic deformity, restrictive lung disease, and impaired bone metabolism.

The close collaboration and information exchange among team members are critical for their success. From geneticists’ analysis of gene genetic variants to surgeons’ surgical techniques and respiratory therapists’ rehabilitation guidance, each link plays a crucial role in the patients’ recovery. This interdisciplinary collaborative model improves treatment effectiveness and reduces postoperative complications, providing essential references for the treatment of similar cases in the future.

In this case, genetic testing was critical for accurate diagnosis. The location of the genetic variants correlated with disease severity: p.Y436C, affecting the catalytic Ca^2+^-binding site, is pathogenic, while p.R71G partially impairs enzyme secretion and is likely pathogenic. This explains the early dental symptoms and delayed progression of chest deformity. Genetic analysis provided precise molecular insight compared to traditional diagnostic methods relying on clinical and biochemical findings. Identifying specific *ALPL* variants enabled both diagnosis and personalized treatment planning. This case highlights the potential of precision medicine in managing rare genetic disorders.

An important aspect of this study was the 16-day postoperative follow-up to assess early surgical outcomes. Compared with other reports, this case provides key perioperative safety data, including wound healing, pain control, and early respiratory improvement (SpO_2_ increased from 85% to 93%). However, this short observation period is insufficient to evaluate the long-term stability of bone remodeling. Recent clinical consensus recommends a standardized follow-up period of at least two years for skeletal correction surgery, with thoracic morphology assessed via 3D CT every six months (40). Additionally, treatment guidelines emphasize the need to monitor both bone metabolism markers (e.g., serum PLP) and functional outcomes (e.g., 6-minute walk test) to assess the durability of surgical intervention (22). Due to the limited follow-up duration, this case has not yet completed such comprehensive evaluations, underscoring the need for extended observation over 3–5 years in future studies.

In summary, this study effectively demonstrates the pivotal role of *ALPL* genetic variants in developing hypophosphatasia and highlights the significance of corrective surgery in ameliorating chest wall deformities and respiratory function through an in-depth analysis of a hypophosphatasia patient. The collaborative efforts of a multidisciplinary team and the utilization of genetic testing were instrumental in diagnosis and treatment, resulting in improved treatment outcomes and valuable insights for future similar cases. However, this study is limited by its small sample size and the absence of data from a broader patient population. Future research should encompass a more significant number of cases to validate the conclusions drawn from this study and explore innovative treatment methods such as gene therapy further to enhance our understanding of and treatment strategies for hypophosphatasia.

## Data Availability

The original contributions presented in the study are included in the article/supplementary material, further inquiries can be directed to the corresponding authors.
