# Supplementary material for: Comprehensive treatment approaches for skeletal deformities in hypophosphatasia: a case study of ALPL gene variants
Source: Front Pediatr. 2025 Jul 9;13:1562878. doi: 10.3389/fped.2025.1562878 (PMC12283614; doi:10.3389/fped.2025.1562878)
Supplement: Supplementary file 1 [file Table1.docx]

**Materials and Methods**

**Genetic Analysis**

This study was approved by the Ethics Committee of the Second Hospital of Lanzhou University (Approval No. [to be inserted]). Peripheral venous blood (3 mL) was collected from the proband and both parents after written informed consent was obtained from legal guardians. Genomic DNA was extracted using the QIAamp DNA Mini Kit (Qiagen, Germany), and DNA concentration was quantified using a Qubit 4.0 fluorometer (Thermo Fisher Scientific, USA). Whole-exome sequencing (WES) was performed on the Illumina NovaSeq 6000 platform (Illumina, USA) with 150 bp paired-end reads. Library preparation was done using the KAPA HyperPlus Kit (Roche, Switzerland), meeting quality standards of ≥100× mean depth and >85% Q30 scores.

Raw sequencing data were aligned to the GRCh38/hg38 human reference genome using BWA-MEM (v0.7.17). Variant calling was conducted with GATK HaplotypeCaller (v4.2.6.1). Annotation was performed using ANNOVAR (2020-06-08), referencing gnomAD (v3.1.1), ClinVar (April 2024), and HGMD Professional (2024.1). Variant classification followed the 2015 ACMG/AMP guidelines. Identified ALPL gene variants were validated by Sanger sequencing using the ABI 3500xl Genetic Analyzer (Applied Biosystems, USA) to confirm familial segregation.

**Biochemical Analysis**

Serum biochemical parameters were analyzed using the Roche Cobas 8000 automated analyzer (Roche Diagnostics, Switzerland). Specific methods included: o-cresolphthalein complexone method for total calcium (reference range: 2.11–2.52 mmol/L), ammonium molybdate UV method for inorganic phosphate (reference: 1.45–2.10 mmol/L for children), and IFCC kinetic method at 37°C for alkaline phosphatase (ALP; reference: 50–350 U/L for children). Liver enzymes ALT and AST were measured by kinetic methods, while γ-glutamyltransferase (γ-GT) was measured by the p-nitrophenol rate method. Internal quality controls were rigorously maintained, and the laboratory participated in national external quality assessments.

**Imaging Evaluation**

Chest wall morphology was evaluated using a GE Revolution 256-slice CT scanner (GE Healthcare, USA), with parameters of 120 kV tube voltage, automated current modulation, and 1 mm slice thickness. 3D reconstruction was performed using Mimics 22.0 software (Materialise, Belgium). Radiation dose was controlled within pediatric ALARA limits (CTDIvol 3.2 mGy, DLP 85 mGy·cm). Standard anteroposterior and lateral X-rays were obtained using a Siemens Ysio Max digital radiography system (Siemens Healthineers, Germany), with exposure settings automatically optimized.

**Surgical Intervention**

The patient underwent a modified Nuss procedure combined with costal cartilage remodeling. A titanium alloy correction bar (Medtronic, USA, model XX-305) was used. General anesthesia was maintained via combined intravenous and inhalational agents, with bispectral index (BIS) monitoring maintained between 40–60. Postoperatively, the patient was transferred to the intensive care unit (ICU) for 48 hours of pressure-controlled ventilation (PCV mode). Multimodal analgesia was administered, including patient-controlled intravenous analgesia (PCIA) and intercostal nerve blocks.

**Follow-Up Evaluation**

Postoperative assessment included both acute-phase (days 1, 3, and 7) and long-term (1, 6, and 18 months) follow-up. Pulmonary function was evaluated using the Jaeger MasterScreen system (Jaeger, Germany), measuring forced vital capacity (FVC) and forced expiratory volume in one second (FEV1). Exercise tolerance was assessed via the 6-minute walk test according to ATS guidelines. Imaging interpretation was performed blindly by two senior radiologists using OsiriX MD 11.0 software (Pixmeo, Switzerland). Parameters such as sternal protrusion angle were measured three times, and intraclass correlation coefficient (ICC) >0.85 confirmed reliability.
